# Individual and environmental factors underlying life space of older people – study protocol and design of a cohort study on life-space mobility in old age (LISPE)

**DOI:** 10.1186/1471-2458-12-1018

**Published:** 2012-11-22

**Authors:** Taina Rantanen, Erja Portegijs, Anne Viljanen, Johanna Eronen, Milla Saajanaho, Li-Tang Tsai, Markku Kauppinen, Eeva-Maija Palonen, Sarianna Sipilä, Susanne Iwarsson, Merja Rantakokko

**Affiliations:** 1 Gerontology Research Center and Department of Health Sciences, University of Jyväskylä, P.O.Box 35, Jyväskylä, FI-40014, Finland; 2 Department of Health Sciences, Lund University, Lund, Sweden

**Keywords:** Life-space, Mobility, Quality of life, Environment, Participation, Physical activity, Walking, Aging, Cohort studies

## Abstract

**Background:**

A crucial issue for the sustainability of societies is how to maintain health and functioning in older people. With increasing age, losses in vision, hearing, balance, mobility and cognitive capacity render older people particularly exposed to environmental barriers. A central building block of human functioning is walking. Walking difficulties may start to develop in midlife and become increasingly prevalent with age. Life-space mobility reflects actual mobility performance by taking into account the balance between older adults internal physiologic capacity and the external challenges they encounter in daily life. The aim of the Life-Space Mobility in Old Age (LISPE) project is to examine how home and neighborhood characteristics influence people’s health, functioning, disability, quality of life and life-space mobility in the context of aging. In addition, examine whether a person’s health and function influence life-space mobility.

**Design:**

This paper describes the study protocol of the LISPE project, which is a 2-year prospective cohort study of community-dwelling older people aged 75 to 90 (n = 848). The data consists of a baseline survey including face-to-face interviews, objective observation of the home environment and a physical performance test in the participant’s home. All the baseline participants will be interviewed over the phone one and two years after baseline to collect data on life-space mobility, disability and participation restriction. Additional home interviews and environmental evaluations will be conducted for those who relocate during the study period. Data on mortality and health service use will be collected from national registers. In a substudy on walking activity and life space, 358 participants kept a 7-day diary and, in addition, 176 participants also wore an accelerometer.

**Discussion:**

Our study, which includes extensive data collection with a large sample, provides a unique opportunity to study topics of importance for aging societies. A novel approach is employed which enables us to study the interactions of environmental features and individual characteristics underlying the life-space of older people. Potentially, the results of this study will contribute to improvements in strategies to postpone or prevent progression to disability and loss of independence.

## Background

Walking, driving and using public transport are the leading forms of mobility among older adults in their local neighborhood. Walking is an integral part of mobility and may be considered a prerequisite for the unassisted use of other modes of transportation. Consequently, the different modes of mobility may share similar risk factors. Mobility is optimal when you are able to go where you want to go, when you want to go, and how you want to go, safely and reliably [1].

The proportion of people over 80 years is growing rapidly. The majority of older people lives in private households and, along with increasing age and declining health, tend to spend more and more of their time inside the home or in its immediate surroundings. Eventually, mobility limitations may render them homebound which, in turn, may lead to marginalization from social activities, loneliness and poor quality of life [2]. A better understanding of the factors that hinder or support the independent community mobility of older people is required to optimize opportunities for active aging and to reduce health disparities.

The disablement model by Nagi [3], later expanded by Verbrugge and Jette [4], outlines the pathway between pathology and disability. Pathology refers not only to physiological abnormalities, such as chronic diseases or injury, but also to physiological changes with advancing age that affect specific body systems and may result in impairments such as strength, balance or sensory impairments. Impairments may lead to functional limitations such as decreased gait speed, which in turn may cause disability. Disability refers to a situation where individual capabilities are not sufficient to meet the requirements of the living environment. The ecological model of ageing, also known as the “Competence-Press model” [5], describes the person-environment relationship in more detail. Environmental factors influence mobility through interaction with the individual’s capabilities, termed person-environment fit (P-E fit). If an individual’s competence and the demands of the environment are in balance, then that individual is able to adapt and function optimally. The ecological model of aging has a strong psychological emphasis, while the disablement process model emphasizes physiological changes. Combining the salient aspects of these two models into an analytical approach may provide a good foundation to better understand age-related changes in mobility.

In the long term, most of the chronic conditions that accompany aging will have a detrimental influence on mobility through various mechanisms involved in the decline in functioning of the musculoskeletal, neurological or cardio-respiratory systems. The impairments most commonly studied in relation to walking difficulties are those that directly influence walking, namely muscle strength and balance. Impairments in sensory functions, such as vision and hearing, also affect mobility [6]. Hearing loss may hinder the ability to divide attention between traffic, having a conversation, maintaining postural balance and walking, thus potentially increasing risk for falls and other accidents [7]. According to one of our previous studies [8], people with co-existing vision and hearing impairments had over four-fold risk, and people with co-existing impairments in vision, hearing and balance almost 30-fold risk, for falls, compared to people with no vision impairment. Falling and fall-related injuries are common among older people, often leading to a sudden and catastrophic disability. Approximately 20% to 40% of community-dwelling individuals older than 65 years fall every year and about half of those who fall do so repeatedly [9]. Falls may lead to progressive mobility decline, even in the absence of consequent injury. In our previous study, older women with indoor falls were over three times more likely to report new difficulties in walking 2 km by the end of the 3-year follow-up compared to those with no falls [10].

Environmental conditions affect outdoor mobility, especially in older adults [11], either by facilitating or restricting participation in out-of-home activities. Environmental barriers for outdoor mobility subjectively reported by older adults include, for example, poor transportation, discontinuous or uneven side-walks, curbs, noise, heavy traffic, inadequate lighting, lack of resting places, sloping terrain, long distances to services and weather conditions [12,13]. Poor street condition, heavy traffic and excessive noise correlate with onset of mobility limitation while pedestrian-oriented designs and access to recreational facilities are positively associated with physical activity and self-rated health in older adults. Similar evidence exists for the association between barriers in the home and difficulties carrying out important daily activities [14]. However, most of the existing studies are cross-sectional. This makes it impossible to know which comes first, the environmental barrier or the mobility problem, and so draw conclusions on causality (for review, see [15]).

Life-space mobility refers to the size of the spatial area (bedroom, home, outside home, neighborhood, town, distant locations) a person purposely moves through in daily life and to the frequency of travel within a specific time and need of assistance for that travel [16]. The first reports on life-space appeared in the aging literature during the 1980s and early 1990s [17,18], but most studies concerned nursing home residents. Life-space mobility reflects the balance between the persons’ internal physiologic capacity and the environmental challenges older adults encounter in daily life. Life-space can be used to evaluate transitions in individuals’ abilities to live independently [16]. Only few studies have addressed life-space mobility in community-living older people [19]. Shrinking life-space probably coincides with giving up valued activities that, maintain the social role of the person, such as visiting friends, participating in out-of-home hobbies, recreation or work, and in general with giving up accessing community amenities, a situation referred to as participation restriction [20]. However, not all older persons with functional limitations necessarily restrict their life-space, if they can find ways to compensate for their difficulties, e.g. by using mobility devices [21]. Consequently, measuring life-space mobility also needs to incorporate compensatory strategies.

## Project aims

### Study aim

The aim of the Life-Space Mobility in Old Age (LISPE) project is to examine how home and neighborhood characteristics influence the residents’ health, functioning, disability, quality of life and life-space mobility in the context of aging.

Specific research questions are:

1. What are the environmental (e.g. distance to services, green spaces, benches by walk ways, heavy traffic, level of urbanization) and individual (e.g. fear of moving outdoors, fear of falling and injury, sensory impairments, walking limitation, personal goals) determinants of life-space mobility, disability and participation restriction?

2. Does life-space mobility correlate with indicators of individual wellbeing (quality of life, depressive symptoms and perceived health)?

3. Do changes (increase, decrease) in life-space mobility lead to parallel changes in indicators of individual wellbeing?

4. What are the environmental and individual predictors of changes in life-space mobility and disability (onset, recovery) and participation restriction?

5. How do facilitating/supporting or encumbering environmental features (perceived and objective) differ according to the extent of life-space mobility or differences in functioning?

6. Do individual and environmental features interact in explaining life-space mobility?

7. What are the individual and environmental factors and their interactions underlying the association of life-space mobility and quality of life in older people?

### Substudy aim

The aim of the substudy is to investigate the relationship between habitual walking activity and life-space mobility among community-dwelling older people with an emphasis on environmental barriers and facilitators to habitual walking activity.

## Methods

### Study design

**Figure 1:**
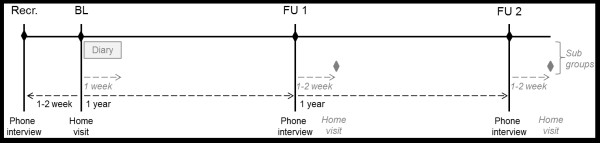
**Conduct of the study.** The diary as well as the home visit at follow-up 1 (FU1) and 2 (FU2) only concern subgroups of participants and are colored gray. Recr. refers to recruitment and BL to baseline assessment.

The study is a 2-year prospective cohort study of community-dwelling older people aged 75 to 90 living in the municipalities of Muurame and Jyväskylä, Finland. Over the 2-year period, four personal contacts will be made with the participants, as shown in Figure 1. The initial contact over the phone was followed by a baseline interview at the home of the participant within 1 to 2 weeks. The baseline home visit will be followed up by phone interviews one and two years later. Subsequently, participants who have relocated since the previous assessment will be visited by a research assistant to gain additional information regarding their relocation and new living environment. The study also provides an opportunity to collect data on the use of health services and on the mortality of the participants from national registers. The substudy on walking activity and life-space mobility was conducted immediately following the baseline assessment.

Muurame and Jyväskylä are neighboring municipalities located in central Finland with total population of 141 500. At the time of drawing the sample, the total population of men and women within that age range was 8 914, of whom 7% lived in sparsely populated areas. For each age group of 75–79, 80–84, and 85–89 years, a random sample of 500 was drawn from the population register on December 12^th^, 2011. The sample was supplemented on March 15^th^, 2012 with an additional 350 persons for each age group to secure a sufficient number of participants. The resulting sample was thus 2 550.

#### Sample size calculations

We calculated that our sample size needed to be at least N = 800 to have sufficient power to also run analyses for subgroups based on age, gender and type of neighborhood. For continuous variables (e.g. life-space mobility variables) a sample size of 800 yields a power of > 99% to show a contribution to the explained variance of 5% in a linear regression model with 10 predictors (including interactions, but not constant) if the probability level (alpha) is set at 0.05. In addition, a sample size of 800 yields even weak correlation coefficients statistically significant. Based on the population characteristics of the recruitment area, 7% of the included population was expected to be living in a sparsely populated area. With a total sample size of at least 800 this would yield a subgroup of about N > 56 persons. In the univariate analyses, correlation coefficients of r > 0.3 would be statistically significant with a two-tailed significance level of 0.05 and a sample size of 56.

### Recruitment process and data collection

#### Contacting participants

A letter containing information about the study including an announcement to expect a phone call the following week was sent to the potential participants. Phone numbers were collected from a nationwide database for all persons in the sample. The research assistant (EMP) called potential participants by phone at the time indicated. When a person could not be contacted, at least 5 repeat phone calls were made on different days and times. When no phone number was available for a person, an information letter was sent including a request to call our research assistant if interested in participating in the study.

### The initial phone interview

During the first phone call potential participants were asked about their willingness to participate in the study and to respond to a short questionnaire with the aim of determining, whether the person was eligible for participation in the study. The inclusion criteria were living independently, able to communicate, residing in the recruitment area and willing to participate. If a person was found eligible, the time for the home visit was set. A letter confirming the time of the home visit was mailed to the participant’s home accompanied by an informed consent form. Persons not interested in participating in the study were asked whether they wished to respond to the same brief phone interview questionnaire as the participants. These data will be used for a non-respondent analysis. The main reason for non-participation (poor health or illness, lack of time, or unwillingness) was recorded if clearly indicated by the person.

#### Baseline face-to-face interview in the participants home

The informed consent form was signed by the participant at the start of the face-to-face interview. The interviews were conducted in the participant’s home using computer-assisted personal interviewing (CAPI). That is, interviewers marked the answers on the electronic forms using laptops. The electronic forms were created using SPSS Data Entry Builder (version 4.0) software from SPSS Inc. A hard copy of the questionnaire was available as back-up in case of computer problems. In addition to the self-report questionnaire, the interviewer objectively assessed the physical performance and functional limitations of the participant and physical environmental barriers in entrances and the immediate outdoor environment. The average duration of the interview was 1.5 hours and the environmental evaluation took approximately 15 minutes.

#### One- and two-year follow-up, phone interview

The first follow-up interview will be made via telephone one year from the baseline interview. When, on the first attempt, a person is not contacted, at least 4 further phone calls will be made to this person on different days and times. If the participant is unable to answer the questions over the phone, the possibility of a face-to-face interview will be offered. For those who have relocated, after informed consent an additional home interview will be conducted by a research team member (JE, MS). The second follow-up interview will be conducted in a similar manner.

### Measures

Age and gender were drawn from the population register as part of the sampling procedure. All the other measures and their time of assessment are listed in Table 1.

**Table 1:** Measures included in the study and the number of items included in each follow-up

**Instrument**	**Domains**	**Assessment**			
		**BL**	**FU1**	**FU2**	**Ref.**
**Main outcome measures**					
Life-Space Mobility (LSA)	Within home	3 items	3 items	3 items	[16]
	Outdoor	3 items	3 items	3 items	
	Neighborhood	3 items	3 items	3 items	
	Town	3 items	3 items	3 items	
	Unlimited	3 items	3 items	3 items	
Quality of life (WHOQOL-BREF)	General	2 items	1 items	1 items	[22]
	Environmental	8 items	8 items	8 items	
	Physical	7 items	-	7 items	
	Social relationships	3 items	-	3 items	
	Psychological	6 items	-	6 items	
**Physical functioning**					
Mobility disability	2 km	1 item	1 item	1 item	[23]
	500 m	1 item	1 item	1 item	
	Stair climbing	1 item	-	-	
	Moving indoors	1 item	-	-	
Pre-clinical mobility limitation	2 km	6 items	6 items	6 items	[23]
	500 m	6 items	-	-	
	Stair climbing	6 items	-	-	
Assistive devices		7 items	-	-	[24]
Short Physical Performance Battery (SPPB)	Standing balance	1 item	-	-	[25,26]
	Walking	1 item	-	-	
	Chair rise	1 item	-	-	
**Physical activities**					
Level of physical activity		1 item	1 item	1 item	[27]
Barriers to physical activity		17 items	-	-	[28]
Avoidance of moving outdoors		2 items	-	-	[29]
Unmet physical activity need		2 items	2 items	2 items	[30]
**Environmental factors**					
Perceived environmental barriers	Outdoors	15 items	-	-	[13]
	Entrance	6 items	-	-	
Perceived environmental facilitators	Outdoors	12 items	-	-	
	Entrance	7 items	-	-	
	Exercise facilities	3 items	-	-	
Type of housing & neighborhood		2 items	-	-	
Objective assessment of environment (HE Screening Tool)	Outdoors	17 items	-	-	[31]
	Entrance	11 items	-	-	
Transportation	Car driving	2 items	2 items	2 items	
	Public transport	4 items	-	-	
	Going to store	2 items	-	-	
**Participation**					
Leisure time activities		5 items	-	-	[32]
Impact on Autonomy & Participation (IPA)	Autonomy outdoors	5 items	-	-	[33,34]
Disability in self-care & instrumental activities	ADL	5 items	-	5 items	[35]
	IADL	9 items	-	-	
	Additional items	2 items	-	-	
**Social context**					
Care giver role		2 items	-	-	
Social contacts & loneliness	Frequency of contacts	3 items	-	-	[36,37]
	Loneliness	1 item	-	-	
	Support	1 item	-	-	
	Marital status & living	2 items	-	-	
Socioeconomic status	Education	2 items	-	-	[38]
	Financial situation	1 items	-	-	
	Profession	1 item	-	-	
	House ownership	1 item	-	-	
**General health**					
Self-rated health & chronic diseases	Self-rated health	1 item	1 item	1 item	[39]
	Chronic diseases	22 items	-	-	
	Weight (loss), height	3 items	-	-	[40]
Cognitive impairment (MMSE)		30 items	-	-	[41]
Depressive symptoms (CES-D)		20 items	-	-	[42]
Sensory functions	Vision	4 items	2 items	2 items	[43]
	Hearing	11 items	8 items	8 items	
Perceived postural balance	General	1 item	-	-	[44]
	Fear of falling	1 item	-	-	
	History of falls	2 items	-	-	
Interviewer-rated functional status		8 items	-	-	[45]
**Personal goals**		1 item	-	-	[46]
**Relocation & major life events**		-	6 items	6 items	

### Main outcome measures

#### Life-space mobility

The main outcome of the project, life-space mobility, was measured with the University of Alabama at Birmingham Study of Aging Life-Space Assessment (LSA) [16], which was translated into Finnish. The translation was done by a back and forward translation procedure by native-speaking English and Finnish translators. Life-space mobility reflects actual mobility performance in daily life. The LSA comprises 15 items and assesses mobility through the different life-space levels (distance), which the participant reports having moved through during the 4 weeks preceding the assessment. For each life-space level (bedroom, other rooms, outside home, neighborhood, town, beyond town), participants were asked how many days a week they attained that level and whether they needed help from another person or from assistive devices. Four indicators of life-space mobility will be calculated [16]: 1) Independent life-space, indicating the highest level of life-space attained without help from any devices or persons, 2) Assisted life-space indicating the highest level of life-space attained using the help of assistive devices if needed but not the help of another person, 3) Maximal life-space, indicating the greatest distance attained with the help of devices and/or persons if needed, and 4) a composite score which reflects the distance, frequency and level of independence (range 0–120). For each LSA indicator, higher scores indicate a larger life-space. Of the LSA indicators, the composite score has the strongest correlation with a person’s observed physical performance [16].

At baseline, life-space mobility was assessed during the face-to-face interview whereas during the follow-ups it will be assessed by phone interviews. The test–retest reliability of LSA scores between a face-to-face interview and a phone interview within two weeks of the baseline assessment was reported to be 0.96 [16].

#### Quality of life

The World Health Organization Quality of Life Assessment short version, WHOQOL-BREF, was used for the baseline assessment of quality of life. It measures individuals’ perceptions in the context of their culture and value systems, and their personal goals, standards and concerns. The 26-item scale comprises four domains; physical health, psychological health, social relationships and the environment. Scoring is calculated separately for each domain and the composite score in all four domains reflects overall quality of life. For participants with one item missing in a subscale or 1–3 items in the total questionnaire, a sum score was calculated (n = 56). In health research, quality of life refers to the general well-being of individuals and comprises wealth, the built environment, physical and mental health, education, recreation and leisure time and social belonging [22].

### Physical functioning

#### Mobility disability and pre-clinical mobility limitation

In the initial phone interview, we studied perceived difficulties in outdoor mobility with the following response categories: 1) able without difficulty, 2) able with some difficulty, 3) able with a great deal of difficulty, 4) unable without the help of another person, and 5) unable to manage even with help. Frequency of going outdoors was studied with the response options 1) daily, 2) 4–6 times a week, 3) 1–3 times a week, and 4) less than once a week. These questions were posed for the purpose of the non-respondent analysis.

In the face-to-face interview mobility disability was studied for perceived difficulties in walking 2 km and 500 m, climbing up 1 flight of stairs and moving around in the home [23]. The response options were similar to those for outdoor mobility. For the four mobility tasks, those reporting that they were able to manage without difficulty were asked about potential modifications in task performance. The presence of the following modifications was determined (yes/no): resting in the middle of performing the task, using an aid, using the support of handrails, having reduced the frequency of performing the task, having slowed down performance of the task, experiencing tiredness when performing the task, or some other change in carrying out the task. These questions identify individuals at an early stage of mobility limitation, that is, preclinical mobility limitation. Preclinical mobility limitation is a state between intact mobility and manifest mobility limitation. Individuals who report task modification have an increased risk of future mobility limitation. However, at the same time they may postpone manifest mobility limitation by making the task performance less taxing by modifying the way they do the task [23].

#### Use of assistive devices for mobility

The use of an assistive device for mobility was rated for seven listed assistive devices with the response options: 1) no, 2) yes, only indoors, 3) yes, only outdoors, and 4) yes, both indoors and outdoors. This list was used previously in the SCAMOB (Screening and Counseling for Physical Activity and Mobility project, ISRCTN07330512) project [24].

#### Short physical performance battery

Lower-extremity physical performance was objectively assessed by the Short Physical Performance Battery (SPPB) [25], which was translated into Finnish [26]. The tests were performed in the participant’s home. The battery comprises three tests that assess standing balance (indicator of balance function), walking speed over a distance of 2.44 meters (general indicator of mobility), and the ability to rise from a chair (indicator of muscle power and strength). Each task is rated from 0 to 4 points according to established age- and gender-specific cut-off points [25]. Participants unable to perform the testing procedure due to mobility-related limitations were assigned a score of 0 for each respective test. Participants unable (e.g. temporary medical condition, wheel chair use, severely impaired sight, lack of suitable a chair) or unwilling to do the tests were assigned a missing score for the respective tests (n = 9 for all tests, n = 3 for one test only). A SPPB sum score was calculated (range 0–12) when at least two tests were completed. When one test was not completed, the maximal score was lowered accordingly. Higher scores indicate better performance. The SPPB is a validated and frequently used tool in older people, with low SPPB sum scores predicting falls, loss of independence and mortality [25].

### Physical activities

#### Level of physical activity

Present level of physical activity was assessed with a self-report scale by Grimby [27] with slight modifications. Both the lowest and the highest category of the initial scale were divided into two categories. The resulting 7-point scale depicted their level of physical activity over the last year: 0) mostly resting, or lying down, 1) hardly any activity, mostly sitting, 2) light physical activity, such as light household tasks, 3) moderate physical activity for about 3 h a week: walking longer distances, cycling and domestic work, 4) moderate physical activity for at least 4 h a week or heavier physical activity 1–2 h a week, 5) heavier physical activity or moderate exercise for at least 3 h a week, and 6) competitive sports. This scale is feasible in older independent populations as it is easy and quick to use and it also rates domestic activities [24].

#### Barriers to physical activity

The questionnaire on barriers to physical activity was developed by an expert panel for our previous study (SCAMOB) and further developed for the present study. The scale includes 17 items under the themes of poor health, fear and negative experiences, lack of knowledge, lack of time and interest, lack of company and unsuitable environment [28]. Each item is rated as yes or no.

#### Avoidance of moving outdoors

Avoidance of moving outdoors was assessed with two questions: “Do you avoid moving outdoors during day time?”, “Do you avoid moving outdoors in the evening?” with response options yes and no. Those responding “yes” were asked the reasons for avoidance. These questions identify persons with fear of moving outdoors [29].

#### Unmet physical activity need

Unmet physical activity need is the feeling that one’s level of physical activity is inadequate, and thus distinct from the recommended amount of physical activity. Unmet physical activity need was studied by the questions “Do you feel that you would have the opportunity to increase your level of physical activity level if someone recommended you do so?” and “Would you like to increase your level of physical activity?” The response options were yes and no. Participants who felt that they had no opportunity to increase their physical activity even though they were willing to do so, were defined as experiencing unmet physical activity need [30].

### Environmental factors

#### Perceived environmental barriers and facilitators for mobility

Barriers and facilitators to mobility in the outdoor and entrance environments of the home were examined as perceived by the participants using standardized questionnaires. The questions on environmental barriers and facilitators were developed by an expert panel for an earlier study (SCAMOB) [13]. For this study, new items were added to this list by an expert group (gerontology, occupational and physical therapy, human geography) with extensive experience in research on environmental effects and health outcomes. Participants were asked whether certain environmental features hindered or facilitated their possibilities for moving outdoors, with the response options yes and no. Altogether, the questionnaire comprised 15 environmental barriers and 12 facilitators for outdoor mobility and six barriers and seven facilitators for mobility in home entrance areas. In addition, three questions about the availability of suitable exercise facilities (outdoor facilities for physical activity, such as walking routes or ski tracks; a park or other green area; and indoor exercise facilities, such as a gym or public swimming pool) within walking distance from the home were asked (yes/no).

#### Type of housing and neighborhood

In the initial phone interview the type of dwelling (apartment block, row house, semi-detached or detached house) was self-reported. At baseline, the interviewer registered the type of dwelling as well as the type of neighborhood (urban, suburban, rural). The neighborhood areas were later confirmed by a researcher from the location on a map.

#### Objective physical environmental barriers

*Physical environmental barriers* in the home and its immediate surroundings constitute an objectively observable factor that can be rated in terms of current standards and guidelines for good housing design in the national context concerned. The environment is described on the basis of standards and guidelines [47] and assessed by professionals.

The Housing Enabler methodology rests on 20 years of methodological development, empirical research and practice application [48]. Since a complete Housing Enabler assessment [49] is complex and time-consuming, we used selected portions of a reduced version; the Housing Enabler Screening Tool [50]. To develop this tool, based on statistical analyses with data from three comprehensive datasets with personal and environmental component data as well as expert panels, the core items of the environmental component of the complete Housing Enabler instrument were identified [31,50]. That is, the physical environmental barriers that are most crucial in relation to the occurrence of functional limitations and the generation of accessibility problems were identified. Whereas the environmental component of an earlier version of the complete instrument [51] contained 188 items, the reduced set had 61. In a subsequent study, this item pool was used as a starting point for creating the Screening Tool. A pilot version was tested for feasibility and inter-rater reliability [50], and after further revisions, the Housing Enabler Screening Tool was established [48,49]. In the LISPE Project, major parts of two of the three sections were used, namely 17 items of the section exterior surroundings and 11 items of the section entrances. The screening involves visiting a dwelling to observe and document the environmental barriers that exist. For three participants the environmental evaluation was not performed since the interview was held in a different location than their home.

#### Transportation

The participants were asked how often they drove a car, travelled by car as a passenger, used public transport such as a bus or a train, and used a taxi or Special Transportation Services. The response options were 1) daily or almost daily, 2) a few times a week, 3) a few times a month, 4) a few times a year, 5) less than once a year, or 6) never. Participants who answered that they never drove a car were asked about their driving history: 1) has never driven a car, or 2) had stopped driving a car. All participants were asked whether they were able to use public transport for daily travelling, and if not, why not.

Participants were asked to report the distance to the grocery store that they commonly use and mode of transportation they commonly used. The response options were 1) on foot, 2) by bicycle, 3) by car, 4) by public transport, 5) by taxi, 6) some other way, and 7) unable to go grocery shopping.

### Participation

#### Leisure time activities

Questions assessing leisure time activities were developed by the research team based on our previous experience in the Evergreen project, a project launched in the 1980s [32]. Commonly reported activities of older people were grouped in a manner relevant for life-space mobility. Activities were categorized based on the setting (inside vs. outside the home) and social context (group vs. individual or small group). In addition, a separate question was formulated on individual physical activities. Participants were asked to rate their frequency of participation in 1) group activities outside the home, such as a choir, physical activity class or church activities, 2) a day care center for older people, 3) individual activities commonly performed inside the home, such as reading, playing music or knitting, 4) individual cultural or other activities outside the home, such as going to a concert, the theater, or a coffee house, and 5) individual physical activities outside the home, such as fishing, berry-picking, walking the dog or gardening. For each question, the response categories were: 1) daily or almost daily, 2) about once a week, 3) about 2–3 times a month, 4) about once a month, 5) several times a year, 6) less frequently, and 7) never.

#### Impact on participation and autonomy

The Impact on Participation and Autonomy questionnaire (IPA) [33] is a validated questionnaire designed to assess perceived autonomy and participation in various clinical as well as older populations. It consists of 5 domains: social relations, autonomy in self-care, autonomy outdoors, family role, and work and educational opportunities. For this study, only the domain “autonomy outdoors” was used. Participants were asked to rate perceived chances in 1) visiting relatives and friends, 2) making trips and traveling, 3) spending leisure time, 4) meeting other people, and 5) living life the way they want. The response categories ranged from 0 (very good) to 4 (very poor). A sum score was calculated, with a higher score indicating more restrictions in participation. For participants with one missing item, the total score was calculated (n = 2). An official Finnish translation was used [34].

#### Disability in self-care activities and instrumental activities

Functional status was assessed using a 16-item self-report questionnaire for Activities of Daily Living (ADL). Basic ADL functions [52] included feeding, rising from or lying down on a bed, dressing, bathing, and toileting. Instrumental ADL (IADL) [53] functions included preparing a meal, doing laundry, shopping, light housekeeping tasks (e.g. doing the dishes), heavier housekeeping tasks (e.g. sweeping the floor), taking medicine, using a phone, using public transport, and handling money. In addition, two new items on the use of cash withdrawal machines and the use of personal computers were added. Participants were asked to rate the ability to perform each task on a 5-point scale ranging from able without difficulty to unable even with help of another person. The questionnaire has been found easy to use and successfully distinguishes older people in different care settings [35].

### Social context

#### Caregiver role

To assess caregiver role, participants were asked whether they were taking care of another person needing assistance in daily life due to illnesses or disabilities. It was specified that the participants could be either formally registered or informal caregivers. Caregivers were also asked whether they lived with the care receiver and to give an estimate of the frequency of care (monthly, weekly, daily, or around the clock).

#### Social contacts and loneliness

The frequency of social contacts was assessed by asking how often the participant met his/her children or other relatives, friends, and acquaintances. The response options for all three questions were 1) daily, 2) weekly, 3) monthly, 4) a few times a year, 5) seldom or not at all, and 6) not having any children or other relatives/friends/acquaintances. These questions have been used previously [36]. Loneliness was assessed by asking whether the person feels lonely, with the response options 1) seldom or never, 2) sometimes, and 3) often. The question was modified from the Evergreen project [37]. In addition, the following question concerning perceived support was asked “Do you have a person that regularly accompanies you when going outdoors and for errands?” (yes or no).

Participants were also asked about their marital status and whether they lived alone or with someone else (a spouse, children, grandchildren, siblings or other relatives) at baseline and in the initial phone interview.

#### Socioeconomic status

Participants were asked to report their highest level of education, their total number of years of education [38] and their most long-term occupation. Participants were also asked to rate their financial situation as 1) very good, 2) good, 3) moderate, 4) poor, or 5) very poor [38]. Home ownership was also asked during the face-to-face interview.

### General health

#### Self-rated health and chronic conditions

Self-rated health was assessed by using a WHO five-point rating scale from very good to very poor [39] in all the assessments, including the initial phone interview. Information on physician diagnosed chronic diseases was collected by self-report using a list of 22 chronic conditions, including different cardiovascular and respiratory diseases, arthritis and diabetes. Furthermore an open-ended question about any other physician diagnosed chronic conditions was used. The method used is similar to the one recommended by Nosikov and Gudex 2003 [54].

Self-reported height and weight were recorded and a question about unintentional weight loss of 5 kg or more was included in the questionnaire. According to previous studies, weight loss has been linked to functional decline and decreased life-space mobility in older people [40].

#### Cognitive impairment

Cognitive impairment was assessed with the Mini-Mental State Examination (MMSE) [41]. The MMSE contains 30 items and the score ranges from 0–30. For those participants who were not able to do one or more parts of the MMSE questionnaire, for example because of blindness, the maximum total score was reduced accordingly (n = 14). A few participants were unable to write, and hence were allowed to dictate the sentence. No reductions in scoring were made because of this.

#### Depressive symptoms

Depressive symptoms were assessed with the 20-item Centre for Epidemiologic studies Depression Scale (CES-D) [42]. The CES-D scale is a widely used self-report measure in community samples. Its reliability and validity has been demonstrated in heterogeneous samples [55]. The participant rated the frequency of each depressive symptom during the previous week. Each item is scored from 0 to 3 with higher scores indicating more depressive symptoms (total score range 0–60). For participants with one missing item, the total score was calculated (n = 15). In the CES-D scale, the cut-off score indicating the presence of clinically important depressive symptoms in community populations is 16 or more [56].

#### Sensory functions

Perceived sensory functions were assessed using a structured questionnaire. Participants’ near and distance vision, with eyeglasses if needed, were evaluated using three questions with three-point rating scales. Furthermore, the interview included a question about possible limiting effects of vision on participants′ mobility [43]. Different aspects of hearing ability as well as hearing-aid usage were assessed by 11 questions. The questions were modified from the Hearing Disability and Handicap Scale and they concerned for example the participants^′^ ability to hear in different listening environments, to locate the sound source and the avoidance of social situations because of the possible hearing difficulties [57,58]. Hearing- and vision-related questions were selected for this study based on the findings of the previous large-scale Finnish studies [44,59,60].

#### Perceived postural balance and falls

Perceived postural balance was assessed with the question: “Are you dizzy or do you suffer from poor balance?” The response options were: 1) rarely or never, 2) sometimes, causing me some distress, and 3) often, causing me much distress. Fear of falling was assessed by the question: “Are you afraid of falling?” The response options were: 1) never, 2) occasionally, 3) often, and 4) constantly [44]. Information on falls (“Have you fallen or slipped during the previous year?” yes/no) and injurious falls (“If yes, how many times you needed physician’s treatment?”) during the previous year was also collected.

#### Interviewer-rated functional status

At the end of the face-to-face interview, an eight-item questionnaire from the Health 2000 project was used for the interviewer-rated assessment of functional status of the participant. The questionnaire includes assessment of mobility, daily activities, vision, hearing, use of hearing aid, ability to talk, ability to understand speech, and temporal disability [45].

### Personal goals

The content of personal goals was studied using a revised version of Little’s (1983) Personal Project Analysis [46]. The following instruction was used in the interview: “We all have different goals that we try to accomplish in our daily lives or achieve in the future. The goals may be related to any life domain, such as hobbies, daily life, health, family or friends. Think about the goals you have in your life at the moment. The goals can be big or small as long as they are important to you”. If the participant did not understand the question, examples of the personal goals of a young person were given. Participants were asked to mention as many personal goals as possible; the number of goals mentioned ranged from zero to seven. These personal goals will be classified by two independent assessors in 22 categories based on their content using a coding scheme developed for the purposes of the study (revised on the basis of the coding scheme used by Salmela-Aro et al. 2009 [61]). Any disagreements between the two assessors will be discussed until total agreement is reached. A participant may have goals in several different categories or several goals in the same category. Eventually, each personal goal category will be coded on a dichotomous scale with zero indicating no goals and one indicating at least one goal in the respective category. Those with no personal goals will be coded separately in a category labeled no personal goals.

Personal goals are defined as states that people strive to achieve or avoid in the future [46] . Research on personal goals among people aged 75+ is limited (e.g. [62]), although setting and pursuing relevant personal goals might affect older adults’ functioning and quality of life in old age.

### Relocation and other major life events at follow-up

At the one- and two-year follow-up interview participants will be asked about major life events since the previous assessment (one year ago) in a semi-structured interview. Major life events, such as those related to health (e.g. new medical diagnoses, worsening or recovery of an existing illness), to mobility (e.g. new mobility problems, or new assistive devises), to the family situation (e.g. death of a spouse) or to living situation (e.g. relocation), and to leisure activities (e.g. new or ended hobbies) may affect life-space mobility.

Participants who have relocated will be asked to give their consent to a short face-to-face interview and evaluation of the entrance and exterior surroundings of their new home with the HE Screening Tool. In this way, we aim to capture changes in the environment and their possible effects on the size of the life-space. The face-to-face interview will also include questions about the reasons for relocation and the related decision making process, and the environmental barriers and facilitators of the new living environment.

### Substudy

A substudy on habitual walking and life-space was conducted in the week following the LISPE baseline face-to-face interview.

### Diary

During the face-to-face interview at baseline, every participant was asked if they would be willing to keep a 7-day diary. Those who agreed were asked to maintain their usual daily activities and record them in a structured diary. Participants were instructed to record the life-space level they moved through each day in accordance with the LSA questionnaire [16]. In addition they listed the places visited. The total time spent outside the home (in minutes) was recorded. In addition, the participants recorded whether unusual events, such as illness or extreme weather, affected their normal daily routines during the day. At the end of the 7-day period, the participants returned the diary to the research center by mail. The life-space diary was developed by an expert group for the current project. Diaries that contained data for more than 4 days and had at most one missing day between two recorded days were considered valid.

### Accelerometer

In the period from March 26^th^ to June 15^th^, 2012, an accelerometer (Hookie, tri-axial, “AM20 Activity Meter”, Hookie Technologies Ltd, Espoo, Finland) was offered to a subgroup of the participants, selected on the basis of interest expressed during the initial phone interview and the availability of accelerometers. The accelerometer was given with detailed instructions provided orally and on paper. In addition to keeping the life-space diary, participants were instructed to wear the accelerometer for 7 consecutive days following the face-to-face interview. They were instructed to wear it over the right side of the waist, over clothing on an elastic belt all day from waking up to the time they went to sleep. It was to be removed only for water activities. Participants were asked to register in their diary the exact time when the accelerometer was put on and taken off. The accelerometer was returned by mail in a foamed envelope. If the participant reported difficulty getting to a post office or mail box, the accelerometer was picked up from the participants’ home.

For the analyses, the default settings for thresholds and formulas for calculating different parameters supplied by the manufacturer will be used. It is possible to obtain the following parameters from the accelerometer: step count, total activity points, average MET (metabolic equivalent of task) and total time spent on different activities (walking, running, low activity and other).

By measuring acceleration (change in velocity with respect to time, m/s^2^) of the body, it is possible to obtain an estimate of the intensity of physical activity. Accelerometers have been shown to be useful in monitoring physical activity patterns of community dwelling older people [63].

### Outdoor temperature

Daily outdoor temperature values were obtained from the local energy company (Jyväskylän Energia Ltd). The daily temperature was a mean value of four measurement points located in different parts of the study area recorded at 1 p.m. Outdoor temperatures were matched with the dates of the diary days.

### Quality assurance

Ten interviewers were recruited from among students in the Department of Health Sciences at the University of Jyväskylä. Interviews were also conducted by two members of the research group (JE, MS). The interviewers received special training before the study started. The training included three days on interviewing techniques suitable for older people, the scales and tests, the criteria for the observational scales, the use of the electronic form for the CAPI, study ethics, safety and the oath of confidentiality. For the objective physical environmental evaluation, the training comprised two days of theoretical and practical training, led by the developer of the HE Screening Tool (SI). A manual of operations was written to standardize the testing procedures.

The CAPI method was used as its advantages include a lower risk for errors and missing data, no separate data entry phase, and availability of data immediately at the end of the data collection phase [64]. The interviewers returned the forms and interview data on a USB memory stick during their weekly visit to the research center. At this time, the material was checked and any unclear issues were discussed. Data saved on the laptop and USB memory stick was blinded in terms of the identity of participants. In addition, we organized a seminar for the interviewers to discuss their experiences during the interviews and to assure the data collection was done according to standard. Throughout the data collection phase members of the research team (MR, EP, EMP, MK) monitored the quality of the data and provided feedback to the interviewers.

### Ethics

The study complies with the principles of good scientific conduct and good clinical practice in all its aspects (Helsinki declaration). The LISPE project was approved by the ethical committee of the University of Jyväskylä, Finland, on November 2^nd^, 2011. The protocol of the study was described in the letter of invitation sent to all the potential participants targeted. During the phone interview and face-to-face interview further information was given and participants were able to withdraw from the study at any point without consequences. Subjects who participated in the face-to-face interview signed a consent form which also included information about the study protocol and measurements. In addition, participants could opt to give permission for the use of register data on health service use and mortality.

### Data management and statistical analysis

#### Statistical analyses

In this study, when inferential analyses are conducted for continuous variables, they will primarily be based on parametric general linear models such as multiple regression and analysis of variance. Markedly non-normally distributed data will be transformed prior to inferential comparisons. For variables that cannot be successfully transformed, nonparametric methods will be used. Categorical variables will be analyzed with logistic regression or categorical response models. If the expected frequencies are too small for asymptotic assumptions, exact testing techniques will be used.

Data will be analyzed using multivariate statistical methods which allow analyses of time-dependent outcomes and covariates. In longitudinal analyses, we will use approaches which take into account mortality as a competing risk for other health outcomes. Person-environment interaction will be analyzed with help of regression analyses. Path modeling and structural equation modeling will be used for multilevel analyses of environmental and individual characteristics relative to the study outcomes. Generalized estimating equations (GEE) models will be used to analyze differences in changes in prevalence over three time points. The level of statistical significance will be set at p < 0.05 (two-sided).

### Data reporting

In all future publications, data will be reported following the STROBE criteria (Strengthening the Reporting of Observational studies in Epidemiology) [65].

## Results

### Study flow and non-respondent analyses

**Figure 2:**
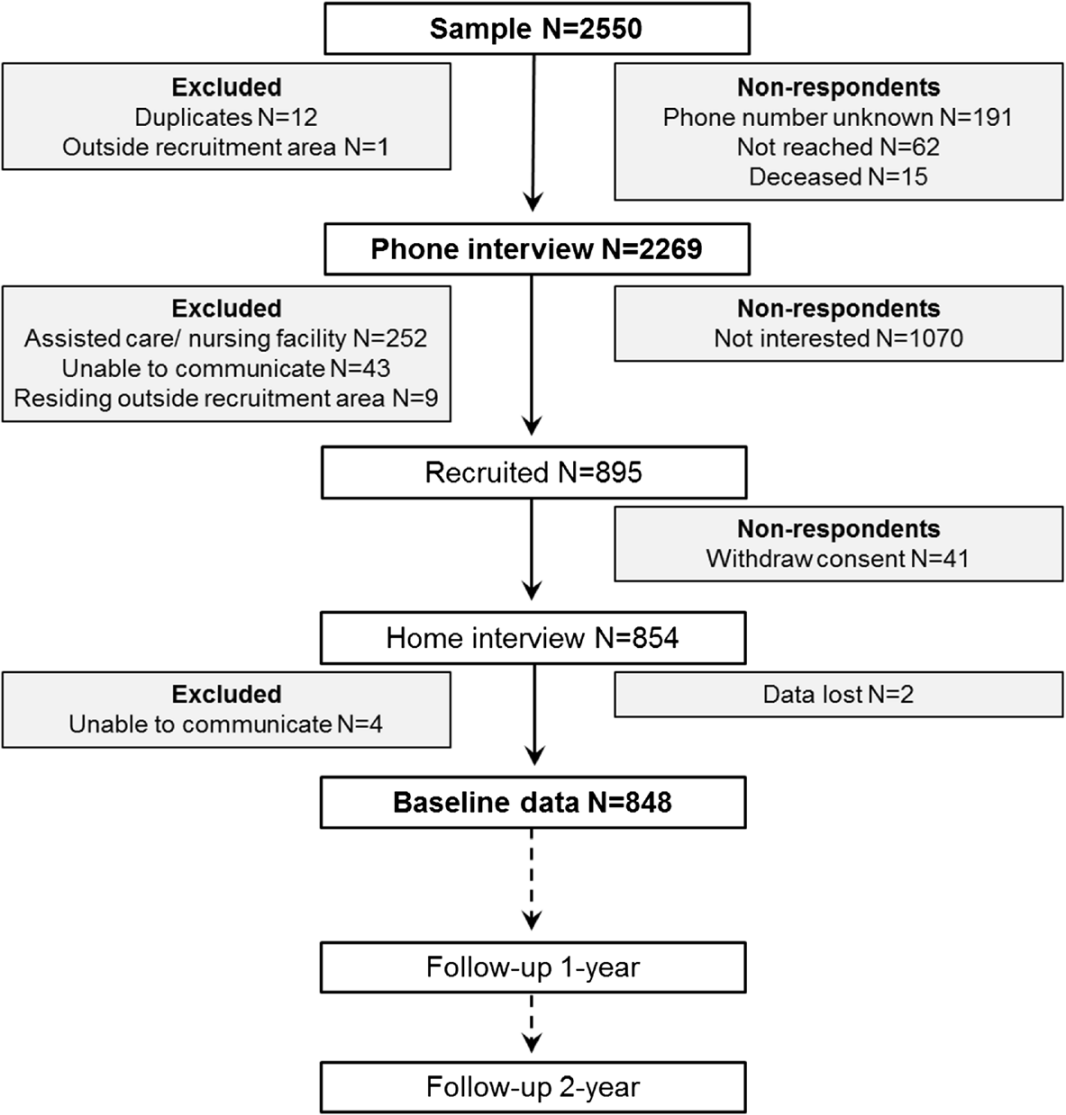
Flow chart of the study.

Figure 2 shows the flowchart of the study. Of the initial sample of 2 550 men and women, 2 269 persons were contacted on the phone to determine their eligibility and willingness to participate in the study. In total, 1 070 persons declined (unwilling n = 551, no time n = 121, poor health n = 398), 304 were not eligible, and 41 persons withdrew their consent between the phone interview and the face-to-face interview. During the face-to-face interview four participants were excluded due to communication problems and the data of two participants were lost due to a technical problem. The total baseline sample size was thus 848. The mean age of the participants was 80.6 ± 4.3 and 62% was women.

In total, 811 of those who declined to participate and 833 of those who participated in the study, responded to the initial phone interview were included in the non-respondent analyses (n = 1644). Those who declined to participate were older (81.2 ± 4.1 vs. 80.4 ± 4.2, Students T-test p < .001), more often lived with a spouse or others (Chi-square test p = .006), more often perceived their health as poor or very poor (p < .001), perceived more difficulties in outdoor mobility (p < .001) and moved outdoors less often than those who participated in the study (p < .001) (Table 2).

**Table 2:** Non-respondent analyses and subgroup analyses

	**Total sample**			**Substudy**			
	**Non-participants**	**Participants**	***Chi-square***	**Accelerometer**	**Diary-only**	**Not in substudy**	***Chi-square***
	(n = 811)	(n = 833)	*P-value*	(n = 170)	(n = 354)	(n = 309)	*P-value*
**Gender** (women)	66.1	62.1	.089	63.5	59.3	64.4	0.367
**Housing type**			.511				0.008
Apartment block	56.7	55.9		49.4	60.7	54.0	
Row house	14.5	16.6		19.4	17.8	13.6	
Semi-detached/ detached house	28.7	27.5		31.1	21.4	32.4	
**Living situation**			.006				0.009
Alone	49.7	53.9		55.3	49.7	57.9	
With spouse	44.2	43.1		44.1	47.7	37.2	
With other person	6.1	3.0		0.6	2.5	4.9	
**Self-rated health**			<.001				0.056
Very good	3.0	5.3		7.1	5.9	3.6	
Good	25.2	32.5		35.2	34.5	28.8	
Moderate	49.9	52.3		52.9	50.3	54.4	
Poor	19.9	9.7		4.7	9.3	12.9	
Very poor	2.0	0.1		0	0	0.3	
**Difficulty in outdoor mobility**			<.001				<0.001
No difficulties	52.5	60.5		67.1	62.7	54.4	
Minor difficulties	30.4	28.9		30.6	28.2	28.8	
Major difficulties	10.6	8.0		2.4	7.1	12.3	
Unable with / without help	6.4	2.5		0.0	2.0	4.5	
**Frequency of outdoor mobility**			<.001				<0.001
Daily	63.5	75.9		84.1	77.1	69.9	
4-6 times/week	15.2	13.0		13.5	13.0	11.0	
1-3 times/week	13.5	8.8		1.8	8.5	12.9	
< once /week	7.9	2.4		0.6	0.3	4.5	

Sample characteristics represented as proportions (%) and tested group differences.

### Substudy flow and non-respondent analyses

**Figure 3:**
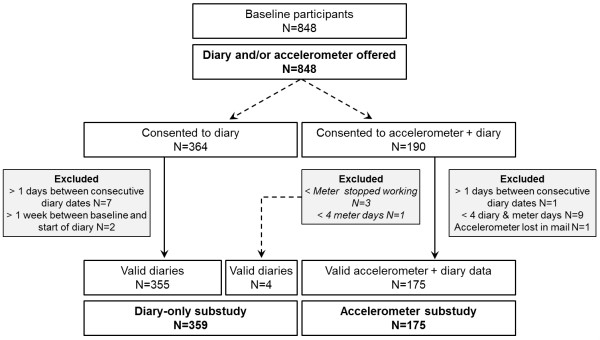
Flow chart of the substudy.

Participation in the substudy on walking activity and life-space was offered to all the study participants during the face-to-face interview. Figure 3 shows the flowchart of the substudy. In total, 364 diaries were returned, of which nine were excluded. In addition, 190 participants were willing to wear an accelerometer in addition to keeping the diary. Of these, 179 participants returned a valid diary. Unfortunately, the accelerometer battery ran out during the measurements in three cases and one case wore the accelerometer occasionally; hence these four were included in the diary-only group. Thus in total, the accelerometer group consisted of 175 participants and the diary-only group of 359 participants.

The participants in our substudy groups were somewhat younger than those not participating in the substudy (accelerometer group 80.4 ± 4.2, diary-only group 79.9 ± 4.1, vs. not in substudy 81.4 ± 4.4, ANOVA p <0.001). In addition, the participants in the substudy groups reported less difficulty in outdoor mobility (Chi-square test p < 0.001) and going outdoors more frequently (p < 0.001). Those participating in the diary-only subgroup lived more frequently in an apartment block (p = 0.008), in addition, they more often lived with another person (p = 0.009). The gender distribution and self-rated health were not significantly different between the three groups. Baseline assessment of level of physical activity showed that 36.6% of those in the accelerometer group and 44.4% of those in the diary only group participated in moderate to strenuous physical activity for at least 4 hours a week or in physical exercise multiple times a week compared to 21.0% of those not participating in the substudy (<0.001).

## Discussion

The purpose of this paper was to present the study protocol of LISPE project. The aim of the LISPE project is to examine how home and neighborhood characteristics influence the residents’ health, functioning, disability, quality of life and life-space mobility in the context of aging.

### Strengths

We were able to recruit a large group of older people in the LISPE project. This means that the study has sufficient power to detect also moderate relations. To obtain a large study population we chose to collect data in personal face-to-face interviews in the participants’ homes. This has the advantage of enabling persons with poorer health to take part. We also know for certain that the intended member of the household was interviewed. In addition, in cases where the participant did not immediately understand the question, the interviewer was able to assist by repeating the question or by providing examples. The home visit also allowed for an objective assessment of the home environment and a performance test in a setting familiar to the participant.

In addition to the face-to-face interview, a large proportion of our participants were willing to participate in our substudy on walking activity and life-space mobility. Compared to previous studies using accelerometer data, our sample size is large. Although the substudy the study participants were somewhat more physically active, the sample also includes older people who reported very little physical activity. In addition, the possibility to combine the accelerometer and diary data with the data collected in the baseline and follow-up interviews, provides a unique research opportunity.

We provided the interviewers with a comprehensive training for their task. The quality assurance system and CAPI method worked well and resulted in quick completion of the analytical datasets. Consequently, the quality of the data is good with very few missing data.

### Limitations

Our goal was to recruit older people with wide variability in mobility. Unfortunately, the non-respondent analysis revealed that those with more mobility problems were more likely to decline participation. Consequently, those with worse mobility are underrepresented in the study. However, this is a common challenge in research among older people [66]. Moreover, we wanted to include participants in different living environments; in the event, we managed to recruit only a limited number of participants in rural areas. Therefore, for this segment of the sample the analyses will be somewhat limited as far as rural dwellers are concerned, although, according to our power analyses, it should still be possible to perform univariate correlations.

We have collected data over a period of six months, starting in winter and ending in summer. The climate in central Finland is characterized by cold winters with ice and snow, and moderately warm summers. Consequently, the extended data collection period may have introduced additional variability in life-space mobility. Although the outdoor temperatures may not affect the lives of the Finnish people too much, people are more likely to stay indoors in winter to avoid walking on slippery surfaces. In this study, it is possible to take outdoor temperature into account. It should also be borne in mind that a wide range of weather conditions are a fact of life in our study area. Consequently, the six-month data collection period may increase the face-validity of the data.

Our participants were rather old at baseline and therefore we tried to restrict the length of the interview so as not to burden them too much. Consequently, some aspects relevant for life-space mobility have possibly been left out of the study. For example, we were able objectively to assess physical fitness only for lower extremity performance.

## Conclusions

A specific strength of this study is that we have collected data on a topic important for aging societies. We have used a novel approach which enables us to study the interactions between environmental features and individual characteristics that underlie the life-space of older people. The results of this study have the potential to contribute to improvements in strategies to postpone or prevent progression to disability and loss of independence.

## Abbreviations

ADL: Activities of Daily Living; CAPI: Computer-Assisted Personal Interviewing; CES-D: Centre for Epidemiologic Studies Depression Scale; HE: Housing Enabler; IADL: Instrumental Activities of Daily Living; IPA: Impact on Participation and Autonomy; LISPE: Life-Space Mobility in Old Age; LSA: Life-Space Assessment; MMSE: Mini-Mental State Examination; SCAMOB: Screening and Counseling for Physical Activity and Mobility; SPPB: Short Physical Performance Battery; WHOQOL-BREF: World Health Organization Quality of Life Scale Short Version.

## Competing interests

Together with dr. B. Slaug, SI is the copyright holder and owner of the Housing Enabler (HE) methodology, provided as a commercial product (see http://www.enabler.nu). The remaining authors declare that they do not have competing interests.

## Authors’ contributions

This manuscript was drafted by all authors. Each author was responsible for writing part of the manuscript and critically revising the complete manuscript. Additional, author contributions were: TR contributed to the concept and design of the study, acquisition of data, and analyses and interpretation of data as the Principal Investigator. EP contributed to the concept and design of the study with specific focus on physical function and activity, acquisition of data and quality assurance, and analyses and interpretation of data. AV contributed to the concept and design of the study with specific focus on sensory function and falls. JE contributed to the design of the study with specific focus on disparity in physical activity, and acquisition of data as interviewer. MS contributed to the design of the study with specific focus on personal goals, and acquisition of data as interviewer. LTT contributed to the design of the study with specific focus on the walking activity, and acquisition of data for the substudy. MK contributed to the design of the study, statistical and methodological support, acquisition of data and quality assurance, and analyses and interpretation of data. EMP contributed to the design and coordination of the study, participant recruitment, and acquisition of data and quality assurance. SS contributed to the concept and design of the study with specific focus on physical and mobility function. SI contributed to the concept and design of the study with specific focus on the environmental assessment, and acquisition of environmental data. MR contributed to the concept and design of the study with specific focus on mobility function, life space and environmental gerontology, acquisition of data and quality assurance, and analyses and interpretation of data. All authors read and approved the final manuscript.

## Pre-publication history

The pre-publication history for this paper can be accessed here:

http://www.biomedcentral.com/1471-2458/12/1018/prepub
